# Photoconversion of Alloreactive T Cells in Murine Peyer’s Patches During Acute Graft-Versus-Host Disease: Tracking the Homing Route of Highly Proliferative Cells *In Vivo*

**DOI:** 10.3389/fimmu.2018.01468

**Published:** 2018-06-27

**Authors:** Katja J. Jarick, Zeinab Mokhtari, Lukas Scheller, Julia Hartweg, Sina Thusek, Duc-Dung Le, Maria Ranecky, Haroon Shaikh, Musga Qureischi, Katrin G. Heinze, Andreas Beilhack

**Affiliations:** ^1^Interdisciplinary Center for Clinical Research (IZKF) Laboratory for Experimental Stem Cell Transplantation, Department of Internal Medicine II, University Hospital, Würzburg, Germany; ^2^Graduate School of Life Sciences, University of Würzburg, Würzburg, Germany; ^3^Rudolf Virchow Center, University of Würzburg, Würzburg, Germany; ^4^Department of Pediatrics, University Hospital, Würzburg, Germany

**Keywords:** T cell migration, acute graft-versus-host disease, mouse models, photoconversion, Dendra2, Peyer’s patch, *in vivo* cell tracking, lymphocyte homing

## Abstract

The regulation of immune cell migration throughout the body is essential to warrant immunosurveillance and to maintain immune homeostasis. Marking and tracking of these cells has proven important to study mechanisms of immune cell trafficking and cell interaction *in vivo*. Photoconversion is a well-suited technique for intravital application because it enables contactless time- and location-specific marking of cells in the tissue without surgically manipulating the microenvironment of the cells in question. However, in dividing cells the converted fluorescent protein may decline quickly. Here, we provide a detailed description of the photoconversion technique and its applicability to tracking highly proliferating T cells from the priming site of T cell activation to peripheral target organs of effector function in a preclinical model. Dendra2^+^ T cells were photoconverted in the Peyer’s patches during the initiation phase of acute graft-versus-host disease (GvHD) and tracked through the mesenteric lymph nodes and the peripheral blood to the small intestine with flow cytometry and intravital two-photon microscopy. Photoconverted alloreactive T cells preserved the full proliferative capacity, homing, and migration of alloreactive T cells in the intestinal lamina propria. We conclusively proved that photoconversion of highly proliferative alloreactive T cells in the Peyer’s patches is an effective tool to study trafficking of alloreactive T cells under physiologic conditions and to GvHD target tissues. This technique can also be applied to the study of immune cell tracking under inflammatory and non-inflammatory conditions.

## Introduction

Immune cells can migrate to distant locations within the body to warrant systemic immunosurveillance. This efficient immune cell homing is a prerequisite to protect against intruders or to regulate immune responses at any given location throughout the body. Specific immune surveillance becomes particularly impressive when comparing the volume of a T cell (35–95 fL) to the dimensions of the entire body and considering the small population size of a particular T cell clone with specific recognition capability (1). T cell homing plays an important role in many immunological reactions—therefore, delineating this process is central for understanding T-cell-mediated immunity. From a therapeutic perspective, it is essential to better understand the underlying mechanism of lymphocyte trafficking. This would allow specific blocking or fostering of homing routes in organ-specific inflammatory conditions such as autoimmune diseases, graft rejection after solid organ transplantation, or graft-versus-host disease (GvHD) following allogeneic hematopoietic cell transplantation.

Pioneering work in tracking of cell dynamics *in vivo* utilized transfer of radioactively labeled lymphocytes into rats, sheep, and other animals (2–6). Detecting radioactivity in different bodily fluids and organs indirectly proved the labeled cells’ presence. Later, the discovery of congenic markers simplified transfer studies because it enabled the detection of adoptively transferred cells *in vivo* without needing to label the cells before transfer (7, 8). The introduction of biocompatible fluorescent dyes permitted tracking of fluorescently labeled, adoptively transferred cells and their division cycle *in vivo* (9). When using cells expressing fluorescent proteins, they require no labeling before transfer or before detection analysis at all (10, 11), and the fluorescence can even be induced at a specific time (12). Still, labeling dyes are widely used as a flexible means to label cells of interest before adoptive transfer (13).

However, none of these labeling methods are time- and site-specific at the same time, therefore it was not possible to mark specific populations of transferred cells after the transfer. Photoconversion is an excellent technique to mark cells *in vivo* in a specific location, because it enables contactless labeling without surgical manipulation of the organ of interest itself. This facilitates studying T cell homing without changing the homing properties of neither the studied cells nor the tissue surrounding the cells of interest. It is pivotal to minimize tissue perturbance and to avoid an experimental bias in the T cell homing process. By contrast, local introduction of a dye may lead to undesirable tissue perturbance and inflammation. Also, this would non-specifically label all cells present. Therefore, photoconversion is an expediant technique to introduce a time- and site-specific label specifically to transferred cells.

To date, several studies have employed photoconversion to monitor immune cell trafficking. The first (14) and most of the following studies employed the photoconvertible protein Kaede. These studies ranged from monitoring subcellular trafficking of single molecules (15) over organelles (16) to whole body trafficking of cell populations. Tracked cells comprised different precursor cells during embryogenesis (17, 18), as well as immune cell populations (19) and pathogens (20). However, there are two limitations when transferring the findings of the mentioned studies to tracking T cells *in vivo*.

First, due to the development of new photoconvertible proteins, there are now even better choices for *in vivo* imaging. Due to its higher pK, the Dendra2 protein is photoconverted 20 times more efficiently than other convertible fluorescent proteins like Kaede and mEOS (21). This renders Dendra2 more suitable for low-illumination and deep-tissue photoconversion. Gurskaya and colleagues derived the photoconvertible fluorescent protein Dendra from the octocoral *Dendronephthya sp* (22). A subsequent A224V substitution in this protein resulted in Dendra2 with improved maturation and brighter fluorescence both before and after photoswitching (23). Photoconversion irreversibly switches Dendra2 green fluorescence from green to red. However, cell metabolism and cell division dilute the converted protein over time, especially in highly proliferating cells.

Second, immune cell trafficking was up to now studied mostly in non-proliferating cells or under steady state conditions (24–26). More limited are studies providing data from infection models (27, 28) or cancer (29), where only a few specific T cell clones divide. Yet, at present, there are neither detailed reports on the photoconversion procedure nor systematic evaluation of possible side effects on T cell functions *in vivo*. Taken together, it remained unclear whether photoconversion would still be applicable to track an immune response with highly proliferative immune cells. Therefore, we tested whether we could employ photoconversion to track alloreactive T cells from the priming site to the peripheral target organ.

Here, we provide the first in-depth description of the intravital photoconversion procedure. We performed *in vivo* mouse experiments to study the homing from the Peyer’s patches as priming sites to the mucosa of the small intestine as target site. We transplanted T cells expressing the photoconvertible protein Dendra2 in mitochondria (16). Subsequently, we photoconverted anatomical sites of initial T cell activation (30, 31) just before trafficking starts, then tracked the cells for the subsequent 24 h to the target site.

We show that photoconverted alloreactive T cells can be tracked from the Peyer’s patches through the mesenteric lymph nodes and the peripheral blood to the small intestines as target site, using flow cytometry. Furthermore, we demonstrate that photoconversion is well suited to observe T cell migration in target tissues with intravital microscopy.

Photoconversion does not impede cell division, homing, and migration in alloreactive T cells. Hence it is a suitable technique to study trafficking of highly proliferative cells *in vivo*. This protocol provides insight into the procedures and caveats of photoconverting alloreactive T cells in the Peyer’s patches during acute GvHD. The photoconversion technique opens new opportunities for effective and exact tracking of cell distribution *in vivo*. It enables researchers to prove directly the time- and location-specific origin of a marked cell in any given tissue analyzed.

## Materials and Equipment

### Materials

Materials are listed in Table 1 corresponding to the experimental procedures.

**Table 1:** Materials used for the photoconversion and associated techniques.

Material	Catalog #	Company
**Dispensables**		

Transplantation		
DPBS without Ca^2+^/Mg^2+^	P04-36500	Pan Biotech
Cell counting chamber (Neubauer)	ZK03	Hartenstein
T cell enrichment kit (CD11b^−^, CD16/32^−^, CD45R^−^, and Ter-119^−^, Dynabeads Untouched Mouse T cells Kit)	11413D	Thermo Fisher
Trypan blue solution		
Enrichment buffer		
Anesthetic solution		
Photoconversion operation		
Eye ointment (Bepanthen^®^)		Bayer
1 ml syringe 26GA × 3/8″ (0.45 mm × 10 mm), BD Plastipak™	300015	Becton Dickinson
1 ml insulin syringe 30Gx × 1/2″ (0.3 mm × 12 mm), Omnican^®^ 100	9151141	Braun
Analgetic (Novalgin^®^)		Sanofi
Pasteur pipettes	2600111	NeoLab
Sterile razors	704028	Body products, Relax Pharma u. Kosmetik GmbH
Sterile dissecting swab (Setpack^®^ size 2)	12780	Lohmann & Rauscher
Sterile gauze swab (Gazin^®^ 5 cm × 5 cm)	13695	Lohmann & Rauscher
Sterile cotton swab (Rotilabo^®^)	EH12.1	Carl Roth
OP towel	800430	BARRIER, Mölnlicke healthcare
70% ethanol	T931.3	Carl Roth
Quickpad^®^ 70% 2-propanol		Holtsch Medizinprodukte GmbH
Povidone iodine (Braunol^®^ 7.5% solution)	3864065	BRAUN
Suture, 6-0 with beveled needle	V301G	Ethicon
Cell isolation		
Sterile scalpel blades, feather #10	BB510	B. Braun
Disposable serological pipettes	760180, 607180, 606180	Greiner Bio-one
Accu-jet^®^ pro	26300	Brandt
Micropipettes	042760930, 642752433, 942741768, 342733754, 042720454, 942711302	VWR
Cell strainer, 70 µm EASYstrainer™	542070	Greiner Bio-one
Tube 50 ml	227261	Greiner Bio-one
Microtubes 1.5 ml	72.706	Sarstedt
5 ml syringe BD Discardit™ II		Becton Dickinson
Cell strainer, 100 µm (MACS^®^ Smart Strainers)	130-110-917	Miltenyi Biotec
Erythrocyte lysis buffer		
Dissociation buffer		
Proliferation assay		
RPMI Medium1640	21875-034	Gibco^®^
Penicillin, streptomycin (PenStrep)	15140-122	Gibco^®^
l-Glutamine	25030081	Gibco^®^
Fetal bovine serum	10270-106	Gibco^®^
Proliferation tracking dye (cell trace violet)	34557	Life Technologies
Anti-CD3 antibody (145-2C11)	553058	BD Pharmingen™
Recombinant human IL-2	589106	BioLegend
Flow cytometry		
U-bottom 96-well plate	83.3922.500	Sarstedt
Anti-CD4 antibody (RM4-5) coupled to APC-Cy7	100526	BioLegend
Anti-CD8 antibody (53-6.7) coupled to PE-Cy7	100722	BioLegend
Viability dye (Zombie aqua)	423101	BioLegend
Cytotoxicity assay		
Black 96-well plate	6005182	PerkinElmer
D-Luciferin, firefly, potassium salt	L8220	BioSynth
Sodium pyruvate, 100 mM, 100×	11360-039	Gibco^®^

**Equipment**		
X-ray irradiation source	CP-160	Faxitron
Surgery tools: 2 fine forceps, 1 pair of small scissors, 1 flexible needle holder, 1 needle holder		Karl Hammacher GmbH and megro
2 heating mats (20 cm × 30 cm)	76085	Trixie Heimtierbedarf GmbH
UV protection glasses	F18P1L051001	Laservision
High-power UV LED lamp, fiber-coupled	Silver LED-405 nm	Prizmatix
Powermeter PM100	discontinued	Thorlabs

**Equipment**		
Glass fiber, 1,500 µm, NA = 0.5		Prizmatix
Collimator, 405 nm, *f* = 4.02 mm, NA = 0.6 SMA fiber collimation	F671SMA-405	THORLABS
Laboratory stand with bosshead and ring clamp		VWR
Infrared lamp	BF 27	Beurer
Thermometer (dual thermo max/min)	E609790	Amarell Electronic
Centrifuge (Megafuge 40R)		Thermo Scientific
Laminar flow hood Hera safe	KS 18	Thermo Scientific
CO_2_ incubators	150i	Thermo Scientific
Water bath	WNB 14	Memmert
FACS Canto II equipped with 405, 488, and 633 nm lasers and a high-throughput sampler (HTS)		Becton Dickinson
Filter sets for FACS 488 channel: 735 LP + 780/60, 655LP + 760LP, 610LP, 556LP + 585/42, 520LP + 530/30, 488/10; 405 channel: 502 LP + 510/50, 450/50; 633 laser: 735LP + 780/60, 685LP, 660/20		
TrimScope II equipped with a titanium sapphire laser (Chameleon Ultra II, Coherent), beam splitters at 500, 570, and 655 nm, bandpass filters 420/50, 535/50, 605/70, and three photomultipliers		Lavision Biotec
IVIS Spectrum	124262	PerkinElmer

**Software**		
FlowJo	Version X	TreeStar
Imaris	Versions 7.7.2 and 8	Bitplane
Living image^®^	Version 4.0	PerkinElmer
Matlab	Version R2016a	Mathworks

**Mice**		
B6;129S-*Gt(ROSA)26Sor^tm1.1(CAG-COX8A/Dendra2)Dcc^*/J	018397	Jackson Laboratories
BALB/c (BALB/cAnNCrl)	Strain Code 028	Charles River
C57BL/6 (C57BL/6NCrl)	Strain Code 027	Charles River

*Materials listed once may be required also for other techniques explained*.

### Preparation of Reagents

– Anesthetic: add 2 ml of Ursotamin^®^ (100 mg/ml, Serumwerk) and 2 ml of Xylavet^®^ (20 mg/ml, CP-pharma) to 21 ml DPBS. Inject 10 µl/g body weight to reach desired concentrations (Ketamin 80 mg/kg, Xylazin 16 mg/kg).
– Analgetics: metamizol (Novalgin^®^): add 266 µl of Novalgin^®^ to 100 ml of drinking water (1.33 mg/ml). In addition, mice may receive 200 mg/kg of Novalgin injection solution. Inject 10 µl of Novalgin injection solution per g body weight, prepared from 8 µl of Novalgin mixed with 192 µl of NaCl, subcutaneously at recovery from anesthesia.
– Erythrocyte lysis buffer: dissolve 89.9 g NH_4_Cl, 10 g KHCO_3_, and 0.37 g EDTA in 1 l of deionized water.
– Trypan blue solution: dissolve 1 g of trypan blue in 100 ml of PBS. Dilute 1:10 in PBS to receive working solution to mix at equal volume with cell suspension.
– Enrichment buffer: dissolve 0.5 g BSA and 0.375 g EDTA in 500 ml DPBS, sterile-filter.
– Dissociation buffer: per mouse, prepare 70 ml. Dissolve 55.836 mg of EDTA in 66.5 ml HBSS without Ca^2+^/Mg^2+^. Add 3.5 ml FCS to receive HBSS with 5% FCS, 2 mM EDTA.
– Proliferation medium: RPMI medium1640 with 10% FCS, 1% PenStrep solution, 30 ng/ml anti-CD3 antibody, and 50 IU/ml IL-2.

## Stepwise Procedures

This protocol focusses on the *in situ* photoconversion technique and subsequent flow cytometry analysis of converted donor T cells in distant sites. Therefore, these sections are in greater detail, whereas additional procedures are outlined in brief.

This study was carried out in accordance with the recommendations and regulations of the Regierung von Unterfranken. The protocol was approved under the protocol number 55.2 2532-2-48.

### Transplantation

#### Donor T Cell Enrichment

T cells were isolated from the spleen of Dendra2^+^ donor mice (16) at the age of 8–12 weeks. Splenocyte suspensions were enriched for T cells according to the manufacturer’s protocol, counted by trypan blue exclusion, and adjusted to a density of 12 × 10^6^ cells/ml in PBS. Typical T cell yields lay between 15 and 30% of the splenocyte input with a final T cell purity of 85–95%.

#### Donor Bone Marrow Isolation

Bone marrow cells were isolated from hind legs (femura and tibiae) of 8- to 12-week-old C57BL/6 mice. Cell numbers were determined by trypan blue exclusion, and the cell concentration was adjusted to 50 × 10^6^ cells/ml. Typical bone marrow cell yields were 1–1.5 × 10^8^ cells per mouse. Both the T cells and the bone marrow preparations can be stored at 4°C for up to 2 h until transplantation.

#### Transplantation

**Figure 1:**
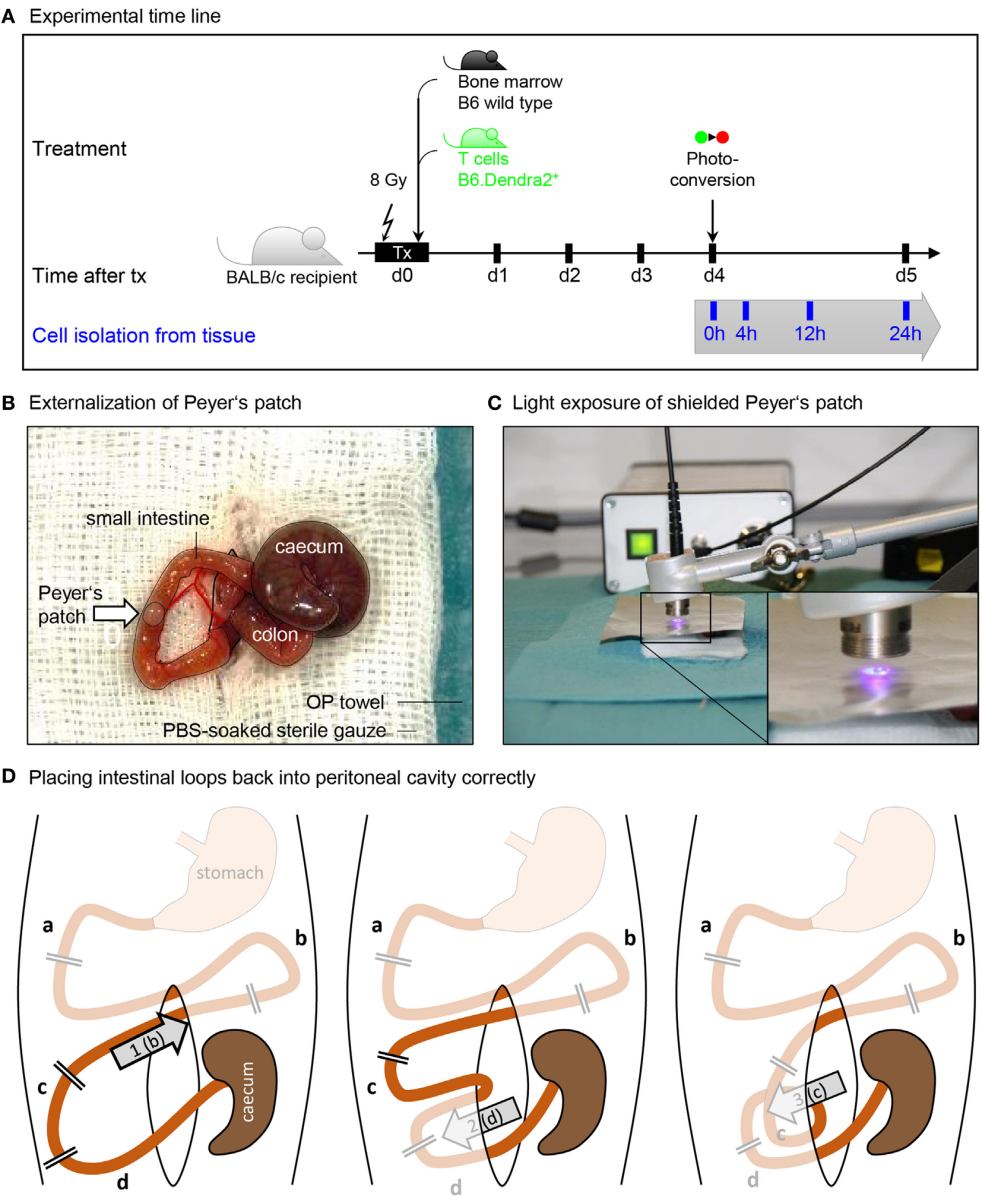
Procedure of Peyer’s patch photoconversion. **(A)** Time line of transplantation and photoconversion procedure. BALB/c recipients were maeloablatively irradiated (8 Gy) and transplanted with wild type bone marrow and Dendra2^+^ T cells. Donor T cells were photoconverted in the Peyer’s patches 4 days after transplantation. **(B)** Photograph of an externalized Peyer’s patch placed on a PBS-soaked sterile gauze. Cecum, colon, peritoneal opening, and small intestine are circled with black lines. **(C)** Application of a tinfoil stencil to expose a Peyer’s patch to UV light illumination using a high-power LED coupled to a glass fiber. **(D)** The duodenum (a) and the stomach-proximal part of the jejunum (b) were not externalized when photoconverting the six distal Peyer’s patches closest to the cecum. After photoconversion, (1) the proximal jejunum has to be placed cranial to the cecum. Subsequently, (2) the ileum (d) is placed opposite of the cecum, after which (3) the distal jejunum (c) is placed ventral of the ileum. Finally, the cecum is placed back at the location where it laid on the gauze.

At the time point of transplantation, recipient mice should ideally have more than 20 g body weight to be sufficiently resilient to partial body weight loss. BALB/c mice at the age of 10–14 weeks were myeloablatively irradiated (8 Gy), and 1.2 × 10^6^ Dendra2^+^ donor T cells were intravenously injected *via* the retro-orbital venous plexus together with 5 × 10^6^ bone marrow cells in a total volume of 200 µl PBS (Figure 1A). The drinking water was supplied with Baytril (Enrofloxacin, 0.05%) for 7 days after transplantation to avoid infections, and mice were monitored twice daily within this period. GvHD was scored clinically and body weight was measured daily.

### Photoconversion

We photoconverted the cells 4 days after transplantation. This is the time point when the majority of donor cells are still in the secondary lymphoid organs and the first donor T cells leave the secondary lymphoid organs to enter the target tissues. This time point is optimal because short intervals between marking the cells and detecting them in the target tissue avoid dilution of the photoconverted protein before detection.

#### Preparation

Two sterile working spaces were prepared: one for animal preparation and one for the operation and photoconversion. For each of them, a heating mat was pre-warmed and covered with a sterile OP towel. All needed instruments and reagents were sterilized and laid out.

An operation cover was prepared by cutting an OP towel to a size of 15 cm × 15 cm, and an oval window was cut into the center with a diameter of 1.5 cm × 2.5 cm to be placed on the operation site.

A gauze swab was prepared by folding it to 7.5 cm × 5 cm and incising a 2 cm long central slit.

The collimator was connected to the UV lamp *via* the glass fiber and fixed on the laboratory stand.

A clean recovery cage was placed under an infrared lamp, and the sensor of a thermometer was placed on the bedding.

#### Operation and Photoconversion

Mice were anesthetized by injecting 10 µl anesthetic solution (Ketamine and Xylazine) per gram body weight intraperitoneally, and placed on the sterile, heated OP towel for preparation. Eyes were protected from dehydration by applying eye ointment. The ventral fur was wetted with 70% ethanol using a dissecting swab and shaved; the skin was disinfected with povidone iodine in an outward circling motion.

After 10 min, anesthetic depth was ensured to be stadium III.2 (surgical tolerance) by pinching the hind paw. If anesthesia was insufficient, further anesthetic was provided retro-orbitally. Not more than 20–50 µl was injected at a time to avoid over-dosage.

The prepared animal was transferred onto a fresh sterile and heated OP towel and the prepared OP-cover was applied. A 1–1.5 cm midline incision was made in the skin and in the peritoneum on the linea alba, no bleeding should be visible. The wound was covered with the prepared gauze swab, the slit overlaid on the incision. The swab was soaked with PBS to prevent drying of externalized intestinal tissue. Next, two cotton swabs were soaked with PBS and used to handle the intestinal contents. The swabs were inserted into the peritoneum to localize and gently exteriorize the cecum, which is located left craniolateral to the incision. The intestinal tissues were handled with great care to prevent a postoperative ileus. First, the cecum was placed on the gauze swab left lateral to the incision, and the intestinal tissue was kept moist at all times by dripping PBS on it using a Pasteur pipette. The small intestine was then gently pulled out, starting from the cecum, until the first Peyer’s patch appeared (Figure 1B). This was placed on the gauze swab facing upwards and covered with sterile tin film. A central hole in the foil with a diameter of 2 mm exposed the Peyer’s patch but covered the surrounding intestinal tissue.

The collimator of the UV lamp was placed at a distance of 5 mm over the Peyer’s patch, and the tissue was illuminated for 2 min at maximum power (Figure 1C). An additional tinfoil shield protected the experimenters from scattered UV light, and UV protection glasses were used whenever the illumination was turned on.

The tin foil was removed, the intestine was gently pulled out until the next Peyer’s patch appeared, and the procedure was repeated until six to seven patches were converted. In order to later be able to identify the converted patches during isolation, the distances in centimeter to the cecum and between the individual converted spots were documented for all converted patches.

After photoconversion, the small intestine and the cecum were gently placed back into the peritoneum as depicted in Figure 1D. Correct organization of intestinal loops was important to prevent a postoperative ileus. The peritoneum was closed with three non-consecutive stitches. Subsequently, the skin was closed with four stitches. The overall operation procedure took 30–45 min per animal. The animal was placed into a clean cage and held warm with an infrared lamp until recovered from anesthesia. The local temperature around the animal was monitored not to rise above 30°C to avoid overheating. When conscious, the animals were transferred back into their group cage. Analgesic was supplied in the drinking water.

### Isolation of T Cells

At different time points after photoconversion (0, 4, 12, and 24 h), mice were sacrificed, and the blood, mesenteric lymph nodes, Peyer’s patches, and small intestine were harvested. One spleen was isolated for control stainings.

#### Peripheral Blood

Mice were anesthetized with Ketamine and Xylazin. After 10 min, the anesthetic depth was ensured to be stadium III.2 (surgical tolerance) by pinching the hind paw. The skin of the animals was disinfected, the animals were fixed on a pad and ventrally opened. The intestine was laid to the animal’s left to expose the left renal vein and the vena cava inferior. An insulin syringe containing 200 µl of lysis buffer was inserted into the vena cava just below the fusion with the renal vein (Figure S1 in Supplementary Material), with the opening facing cranial and ventral. Then, the needle opening was turned to the animal’s left and gently pushed to the ventral side of the vena cava. Blood was collected very slowly to prevent collapsing of the vessel. Up to 700 µl were collected per animal. The erythrocytes were lysed in 30 ml of lysis buffer for 10 min at room temperature. The lysis was stopped by adding 20 ml of DPBS. The cells were pelleted for 5 min at 330 × *g* at 4°C and resuspended in 5 ml of PBS. All processed cell suspensions can be stored for up to 4 h at 4°C in DPBS until further analysis.

Afterward, the mouse was killed by cervical dislocation. The intestinal tract was removed from duodenum to rectum and placed on PBS-moistened paper towels. The mesenteric lymph nodes were excised, and all converted Peyer’s patches were excised generously and trimmed afterward. All non-converted Peyer’s patches were removed from the small intestine.

#### Mesenteric Lymph Nodes

All surrounding tissue (fat and vessels) was carefully removed from the mesenteric lymph nodes. The lymph nodes were kept in a 1.5 ml tube containing 200 µl of PBS for up to 30 min on ice until further processing.

#### Peyer’s Patches

Peyer’s patches surrounded by intestinal tissue were spread out on PBS-moistened paper towels, and all surrounding tissue was precisely cut off using a scalpel. Exact trimming was fundamental for a good T cell recovery. The patches were kept in a 1.5 ml tube containing 200 µl of PBS for up to 30 min on ice until further processing.

Mesenteric lymph nodes and Peyer’s patches were thoroughly minced through a PBS-pre-wet 70 µm strainer using the sterile plunger of a 5 ml syringe, and rinsed through with 5 ml of ice-cold PBS. Cells were pelleted at 330 × *g* for 5 min at 4°C, and the supernatant was removed. Lymph node cells were resuspended in the residual volume and directly transferred completely onto a 96-well plate for staining. Cells from the Peyer’s patch were washed once with 5 ml of ice-cold PBS, centrifuged, and then transferred onto the 96-well plate in the residual volume.

#### Intestine

Cells were isolated from the intestine using a protocol modified from Geem and colleagues (32). The small intestinal tube was opened longitudinally and vigorously dragged through 300 ml of PBS to remove fecal contents. It was cut into 2 cm segments and transferred into a 50 ml tube containing 30 ml of dissociation buffer. The tube was horizontally fixed on an orbital shaker set to 250 rpm for 20 min at 37°C. The solution was passed through a 100-µm strainer into a 50 ml tube. The incubation and filter step were repeated with another 30 ml of dissociation buffer. The first dissociation fraction was centrifuged at 330 × *g* for 5 min at 4°C and the pellet was washed in 30 ml of PBS. The two filtered cell suspensions were united and the PBS washing step was repeated. In initial experiments, the lamina propria fraction was additionally processed according to Geem et al. (32), but all donor T cells were found in the epithelial wash fraction (Figure S2 in Supplementary Material).

#### Spleen

A 70-µm cell strainer on a 50-ml Falcon was pre-wet with 2 ml of erythrocyte lysis buffer. The organ was placed on the strainer, incised crosswise a few times, minced with the plunger of a 5-ml syringe, and passed through the strainer by rinsing with 8 ml of erythrocyte lysis buffer. Erythrocytes lysis was stopped after 2 min by adding 10 ml of DPBS through the strainer. Cells were pelleted for 5 min at 330 × *g* at 4°C and resuspended in 5 ml of PBS.

### Flow Cytometry

Up to 1 × 10^6^ cells were stained per well. Cells were resuspended in 100 µl blocking buffer and incubated for 5 min at 4°C. 100 µl of antibody mix was added, and cells were stained for 30 min at 4°C in the dark. Cells were pelleted at 330 × *g* for 5 min at 4°C and resuspended in 200 µl of PBS. The stained cells can be measured up to 6 h after staining when stored in the dark at 4°C. Longer pauses may require preceding fixation of the stained cells.

Fluorescence signal was acquired at 1,000–3,000 events/s, and washing wells were measured between different sample groups to exclude cross-contamination. The Dendra2 signal is extremely bright, especially in the FITC/GPF channel, and care was taken not to saturate any PMT. Table S1 in Supplementary Material contains a representative compensation matrix.

Donor T cells were identified by gating on lymphocytes, live cells, singlets, Dendra2-green, and/or Dendra2-red positive cells (Figure S3 in Supplementary Material).

### Proliferation Assay

Splenocytes were isolated from Dendra2^+^ mice as described in Section Spleen. Half of the cells were photoconverted in a black 24-well dish for 2 min with a rotating motion of the glass fiber and without the collimator. 5 × 10^6^ cells/ml were stained with 1 µl of cell trace violet for 6 min at room temperature. 500 µl of FCS and 5 ml of RPMI were added successively, and samples were incubated at 37°C in a water bath for 5 min. The samples were pelleted at 330 × *g* for 5 min at room temperature and resuspended in 5 ml of PBS. Cells were seeded on a 96-well round bottom plate at 200,000 cells/well in 200 µl of proliferation medium and left to proliferate for 3–4 days.

### *In Vivo* Cell Migration

Mice were anesthetized and an intestinal loop was exposed as described in the photoconversion operation procedure (see Operation and Photoconversion). Instead of wetted gauze and OP towels, cling foil was used to avoid lint sources in the microscope. The mouse was positioned on a heating pad and the intestinal loop was positioned under a glass cover slip using two custom-made holders. The tissue was kept moist using sterile 0.9% NaCl solution. Fluorophores were excited at a wavelength of 840 nm. The light intensity was increased as the square of penetration depth along the *z*-axis, between 5 and 30%. Images were acquired every 30 s in a field of view of 495 µm × 495 µm with a resolution of 256 × 256 pixels and a distance of 3 µm between two z-planes.

### Cytotoxicity Assay

Lymphocytes were isolated from the axillary, brachial, cervical, inguinal, and mesenteric LN and the spleen of naïve Dendra2^+^ mice. The containing T cells were stimulated in a mixed lymphocyte reaction (MLR) with allogenic BALB/c splenocytes that had been irradiated with 30 Gy or that had been T-cell-depleted. 8 × 10^6^ B6; Dendra2^+^ responder cells were incubated with 4 × 10^6^ BALB/c stimulator cells in 2 ml/well of RPMI with 10% FCS, 1% Pen/Strep, and 1 mM sodium pyruvate in 24-well plates for 4–5 days at 37°C, 5% CO_2_. Cytotoxicity was assessed in round bottom 96-well plates with 50,000 Luciferase-expressing MOPC-315.BMP2.FUGLW target cells (33) and 50,000 to 5 × 10^6^ MLR effector cells containing cytotoxic T lymphocytes. Target cells only were used as a negative control, 0.1% Triton was used as a positive control. Survival of the target cells was quantified from the bioluminescence signal. The cells were pelleted at 330 × *g* for 5 min and resuspended in 110 µl of 2.75 mg/ml Luciferin in PBS. The cells were incubated for 3 min at room temperature and the signal was measured in a black 96-well plate. Percent of lysis was calculated with the formula (experimental signal − background signal)/(maximal signal − background signal) × 100.

## Results

### T Cells Are Photoconverted in Peyer’s Patches

**Figure 2:**
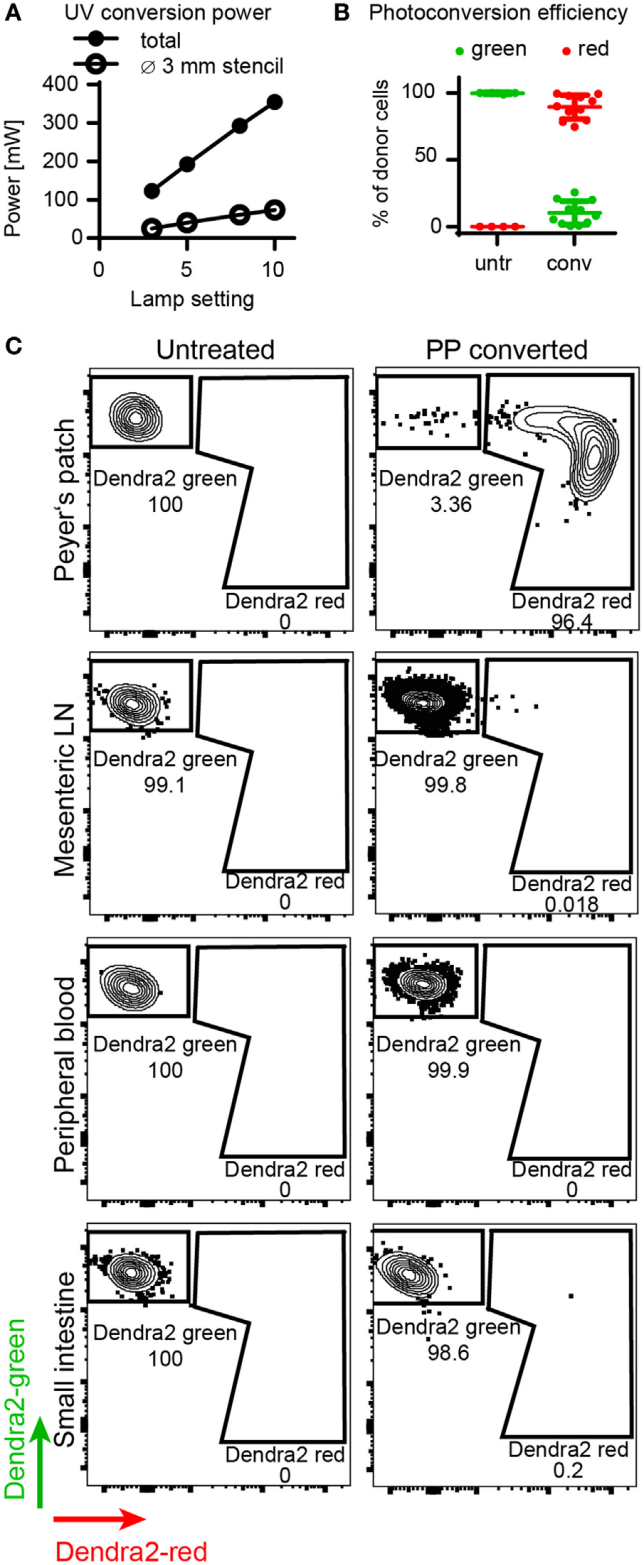
Efficiency of Dendra2^+^ donor cell photoconversion in the Peyer’s patches. **(A)** Output power of LED lamp as measured by power meter. The plot shows total output (without stencil) and applied power on Peyer’s patch (with stencil). **(B)** Photoconversion efficiency of donor T cells in the Peyer’s patch. Before photoconversion, 0% of the cells contained red protein, after photoconversion, 89% (mean) of the cells were red. **(C)** Representative FACS plots of donor cells isolated from the Peyer’s patches, mesenteric lymph nodes, peripheral blood, and the small intestine before and right after photoconversion. Peyer’s patches contain a clear photoconverted population after photoconversion, whereas all other organs contain unconverted cells at this time point.

First, we determined the efficiency of *in situ* photoconversion of Dendra2^+^ T cells in the Peyer’s patches. The LED UV lamp transferred 73 mW of UV light onto the Peyer’s patch through a stencil with a diameter of 2–3 mm (Figure 2A). The *in situ* photoconversion efficiently switches the majority of Dendra2^+^ donor cells in the Peyer’s patch from green to red. About 90% (range: 75–98%) of donor cells isolated immediately after photoconversion contained photoconverted Dendra2 protein (Figure 2B). We detected photoconverted cells only in the Peyer’s patches, and no photoconverted cells leaked into the blood or the small intestine at this time point (Figure 2C). Only single cells resided in the mesenteric lymph nodes immediately after the photoconversion procedure, which took up to 30 min per mouse. In conclusion, photoconversion of the Peyer’s patches reliably marks the majority of currently residing Dendra2^+^ donor T cells in the converted tissue area.

### T Cells Retain Their Proliferative Capacity

**Figure 3:**
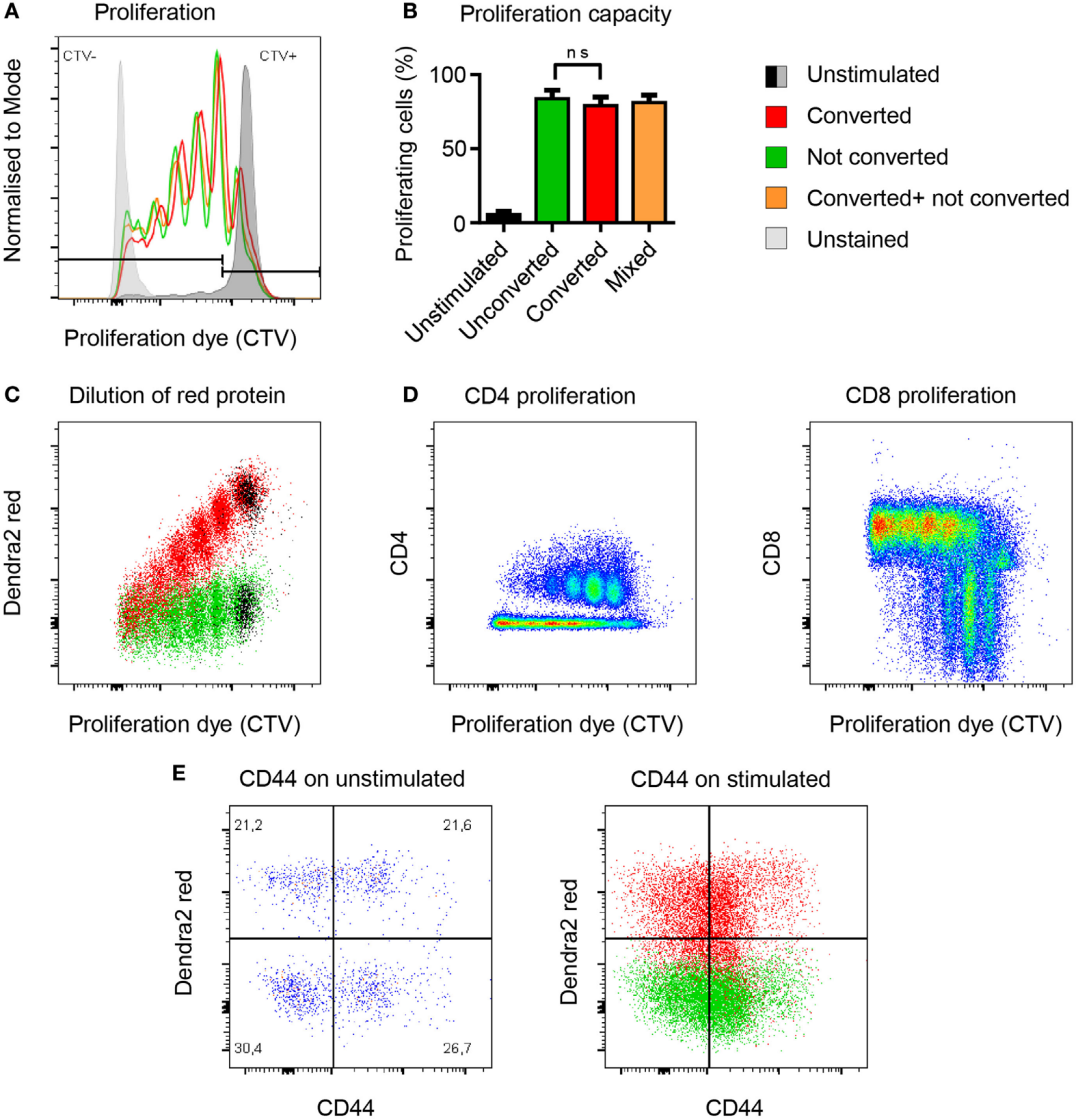
Photoconversion does not impact the proliferative capacity of Dendra2^+^ T cells. **(A,B)** Converted (red), unconverted (green), and mixed (yellow) cells proliferate equally well (non-parametric unpaired test, Mann–Whitney). **(C)** Flow-cytometric signal of the photoconverted protein plotted against the proliferation dye. Each cell division leads to a 50% reduction of fluorescence intensity of the photoconverted fluorescent protein Dendra2-red. **(D)** Comparison of CD4 and CD8 cell proliferation upon polyclonal activation of photoconverted Dendra2^+^ T cells. More rapidly dividing CD8 T cells lose faster their fluorescent cell trace violet (CTV) signal because they proliferate more than the CD4 T cells under this *in vitro* condition. **(E)** CD44 expression as a marker of T cell activation before and after stimulation. CD44 levels are comparable in photoconverted cells (red) versus not photoconverted cells (green). All exp: (*n* = 3 mice).

To address whether photoconversion of T cells would affect key biological functions such as T cell proliferation, we compared the proliferative capacity of photoconverted T cells versus untreated T cells *in vitro*. Both populations proliferated to the same extent (Figures 3A,B). To find out how long the photoconverted Dendra2-red remains detectable in rapidly dividing cells, we measured the decay of red fluorescent signal during proliferation. Stimulated splenocytes lost the photoconverted Dendra2-red signal at the same rate as the proliferation dye, with half of the red signal being retained after each division cycle. The photoconverted population separated from the untreated control for at least four divisions before the two populations merged (Figure 3C). In this setting, CD8 T cells had completed two to five or more cycles and proliferated more vigorously as compared to the CD4 T cells, which had divided one to four times after 4 days proliferation (Figure 3D). We measured the activation marker CD44 before and after stimulation on converted versus unconverted T cells. Photoconversion did not affect the expression of CD44 with or without stimulation after 4 days in culture (Figure 3E). Overall, photoconversion does not affect the cells’ ability to proliferate. Notably, non-dividing photoconverted Dendra2-red^+^ T cells retained their fluorescence intensity at least 4 days in cell culture.

### T Cells Traffic From Peyer’s Patches to the Mesenteric Lymph Nodes, Blood, and Intestine

**Figure 4:**
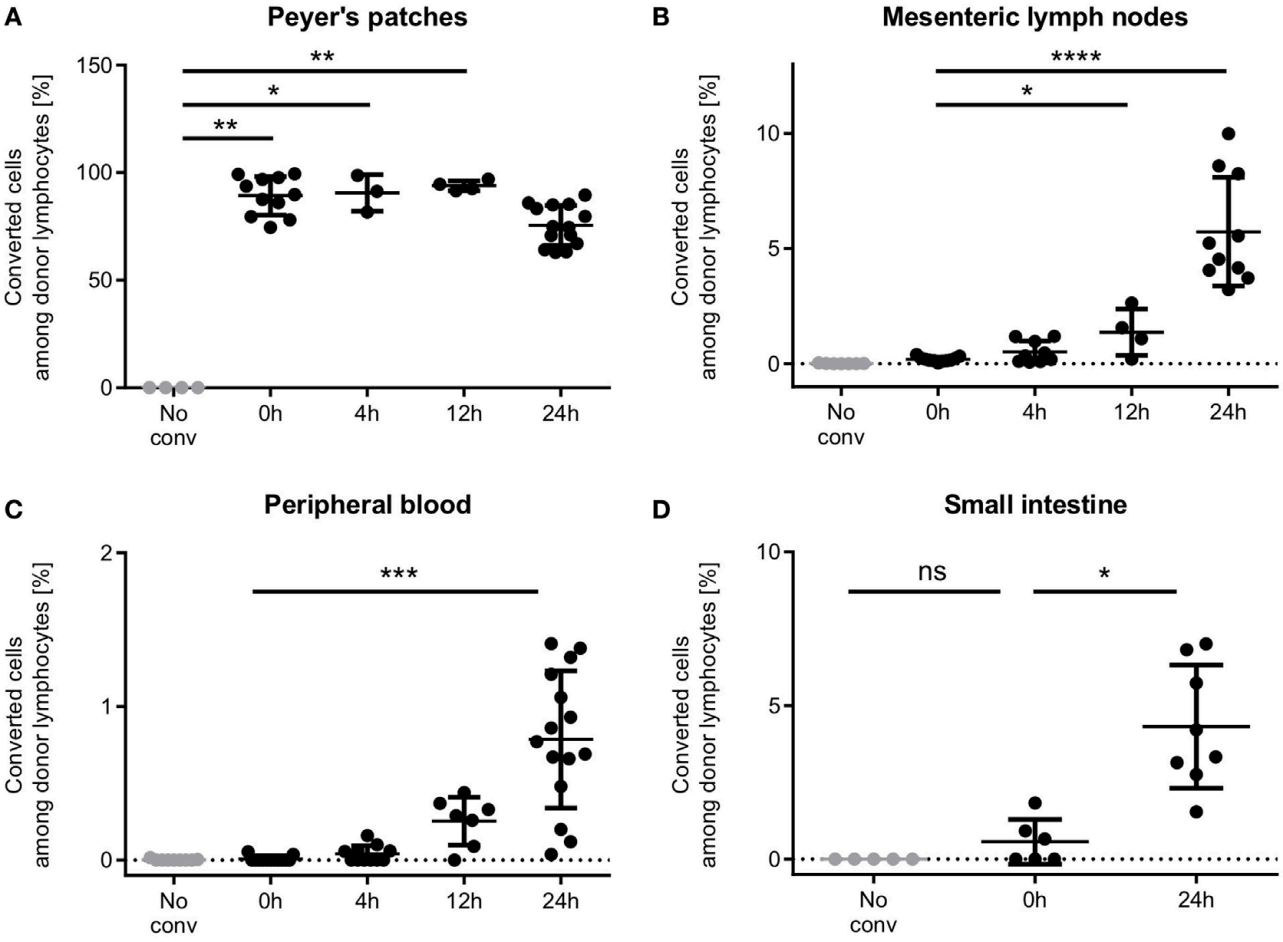
Time course of T cell homing from Peyer’s patches through the mesenteric lymph nodes and the blood to the small intestine. **(A)** Peyer’s patches contain high percentages of photoconverted cells within the 24 h period observed. **(B)** Single cells enter the mesenteric lymph nodes during the photoconversion procedure, and low numbers populate the mesenteric lymph nodes as early as 4 h after photoconversion. 24 h after photoconversion, on average 5.7% of donor cells are from the photoconverted Peyer’s patches. **(C)** First photoconverted cells appear in the blood after 4 h and increase to an average of 0.78% (range: 0.04–1.41%) 24 h after photoconversion. **(D)** Cells were readily detected in the intestine 24 h after photoconversion. In some mice, single red cells show up in the intestinal sample, presumably due to contamination. One data point represents one mouse. Statistics are according to an unpaired non-parametric Kruskal–Wallis test, *p < 0.05, **p < 0.01, ***p < 0.001, and ****p < 0.0001.

To evaluate whether photoconversion would affect T cell motility or efficient homing, we analyzed the homing behavior of photoconverted Dendra2^+^ donor T cells at different time points after photoconversion (Figure 4). After 4 h, first T cells had left the Peyer’s patches and resided in the mesenteric lymph nodes, where 0.5% of donor lymphocytes originated from the photoconverted Peyer’s patches. In the peripheral blood, only 0.04% of donor lymphocytes originated from the Peyer’s patches.

12 h after photoconversion, cell numbers had increased in both sites, mesenteric lymph nodes and peripheral blood, as compared to 4 h after photoconversion, and reached even higher numbers after 24 h. Notably, the vast majority of donor T cells in the Peyer’s patch still remained positive for the red fluorescent signal at this time point. We readily detected T cells in the small intestine after 24 h at a frequency of 4.3% (mean).

### Photoconversion Does Not Impair T Cell Migration Within the Target Organ or Cytotoxicity

**Figure 5:**
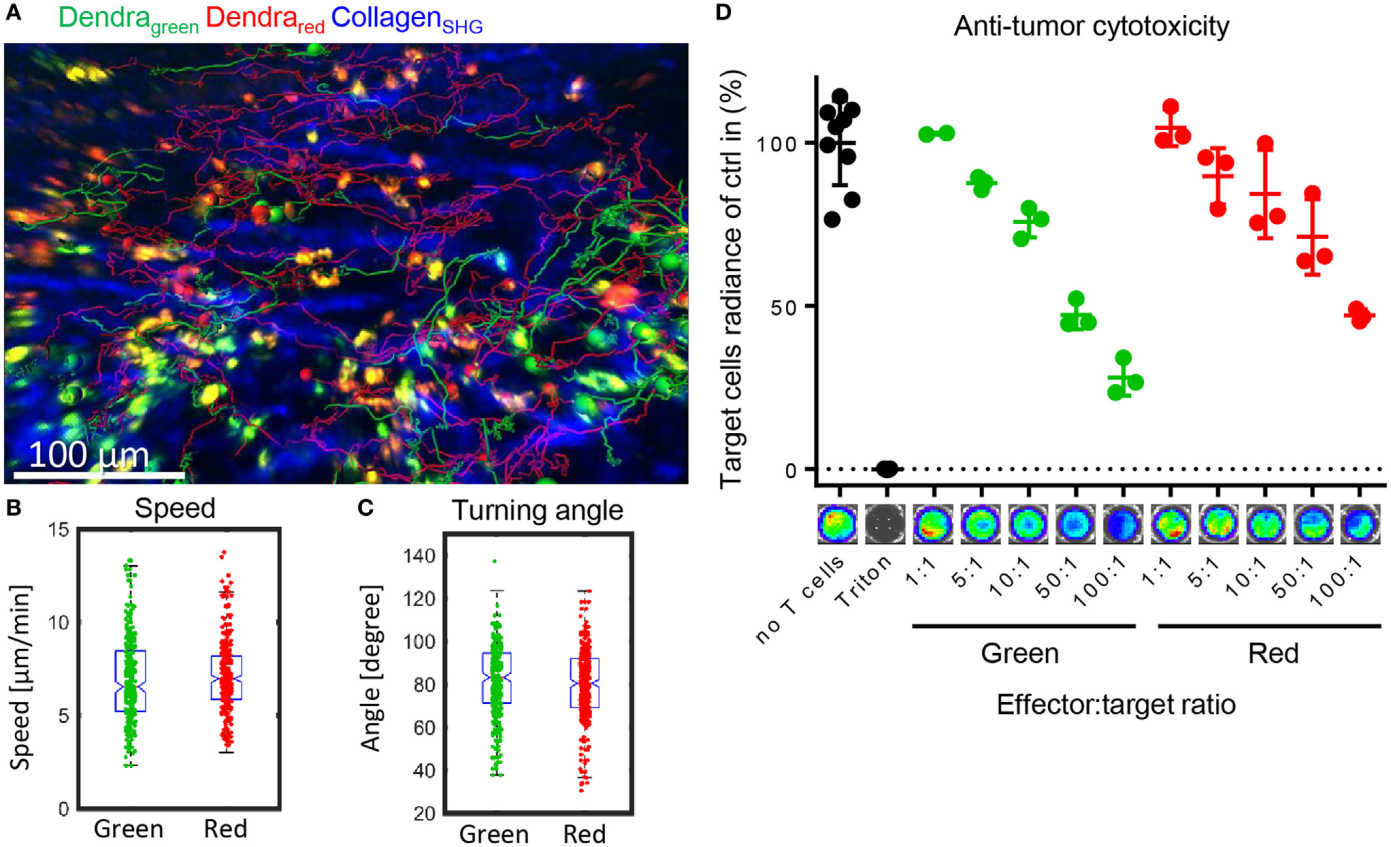
Photoconverted T cells migrate physiologically in the target organ small intestine. **(A)** Representative tracks of green and red Dendra2^+^ T cells in the lamina propria of the small intestine, a graft-versus-host disease target organ. Data were collected with intravital two-photon microscopy. **(B)** Similar migration speed as a basic measure of motility and **(C)** similar turning angles as a basic measure of directionality (both Student’s *t*-test: ns) in green and red cells in the lamina propria (*n* = 266 green and 419 red cells). **(D)** Cytotoxicity assay measuring Luciferase activity of target BALB/c tumor cells. Allogenically stimulated Dendra2^+^ cells were photoconverted or not and then co-incubated with target cells for 6 h. Red and green cells lysed to a similar extend (*n* = 3, Dunn’s multiple comparisons test: ns).

After entering the target organs by extravasation, alloreactive T cells migrate in the tissue to find their target cells and mediate the potentially lethal graft-versus-host response. We tested whether the photoconverted T cells performed equally well in migrating in the target organ, and found that the photoconverted cells were able to migrate as well as the green cells in the lamina propria of the small intestine (Figure 5A). Migration speed as a basic measure of the ability to migrate, and the distribution of turning angles as a measure of directionality were not affected by photoconversion (Figures 5B,C). Furthermore, we assessed whether photoconversion affects the cells’ cytotoxicity. The photoconverted cells performed similarly in lysing the target cells *in vitro* (Figure 5D). These results indicate that homing, migration, and effector functions of alloreactive T cells are preserved after photoconversion. We can track the cells on their way from the priming to the target site without perturbing the course of the immune reaction.

In summary, photoconverted T cells fully retained the ability to proliferate, to traffic to distant sites, and to migrate within tissues at a time point between the initiation and effector phase of acute GvHD. Therefore, we conclude that photoconversion is suitable to study adaptive immune responses such as the homing and migration of heavily proliferative alloreactive T cells in GvHD.

## Discussion

Our findings demonstrate that Dendra2-expressing T cells can be efficiently photoconverted in the Peyer’s patches during the induction phase of acute GvHD. Photoconversion yields proliferating cells that traffic *via* their regular homing route to the intestine. There, they migrate within the tissue to find their target antigen. Hence, photoconversion proved to be applicable to this highly proliferative cell population for at least four cell divisions. The main advantages of this technique are

- Contactless and efficient labeling of cells.
- Effective and exact tracking of cells *in vivo*.
- Direct evidence about time and location of origin in any given organ analyzed.

We photoconverted the cells toward the end of the initiation phase. This is the time when the first cells start entering the small intestinal lamina propria, and therefore, it is a suitable time point to track the early effector phase in the target organ. At the time point of photoconversion, more than 90% of cells in the Peyer’s patches express CD44 and the majority have divided (34). We readily detected the first infiltrating cells in the lamina propria 24 h after photoconversion, which corresponds to day +5 after transplantation. At this time point, the donor cells had a larger size than when transplanted as measured by flow cytometry (data not shown), indicating a T cell blast phenotype. Since at comparable time points, almost all donor T cells proliferated (35), one can expect that more than 90% of the cells detected have undergone multiple divisions. This means that low numbers of proliferating cells can be detected with high specificity. Care has to be taken to exclude cell debris and dead cells in order to receive results that are biologically relevant (see also Figure S3 in Supplementary Material). Although there is a contamination of other cell types in the transplanted enriched T cell population (5–15%) which will also be photolabeled if present in the Peyer’s patches, the vast expansion of the donor T cells in an allogenic setting outgrows the contamination by far. Furthermore, contaminating non-immune cells will most likely not home to the host lymphoid organs after transplantation. Therefore, we expect that all donor cells detected 5 days after transplantation are indeed donor T lymphocytes. The majority of these cells is still positive for the photoconverted fluorophore at the time point of detection 24 h after photoconversion. Therefore, later time points during the effector phase are likely to yield similarly positive results, since more cells will leave the Peyer’s patches during the course of disease.

It is not possible to determine the maximum time after conversion that converted cells remain detectable in the intestine. This is because the mice die from GvHD about 4 days after photoconversion (about 8 days after transplantation) in this model. Nevertheless cells proliferate greatly in GvHD. It is thus likely that the photoconversion signal would be diluted out after five division cycles in these cells (Figure 3). This is expected to be 24–48 h after photoconversion assuming a division rate of 6 h (36). This is distinctly different from the lack of dilution of photoconverted Dendra2 protein in a non-proliferative setting. In such steady state circumstances, it is possible to follow the fate of photoconverted cells for several months (28). A further problem arises if the cells to be converted are in large or highly pigmented organs. The converting light may then not penetrate deeply enough. Hence, it is necessary to ensure efficient penetration by the converting light before performing homing analyses to grant complete conversion of the residing cells. Furthermore, highly pigmented organs heat up more than normal tissues under the UV illumination. In such organs, careful measures are needed to avoid tissue overheating and drying. Extended troubleshooting guidelines are displayed in Table 2. More factors may have to be adjusted when using other photoconvertible proteins which photoconvert less efficiently. Finally, there were no visible alterations of the microenvironment, but we cannot exclude minor phototoxicity to cell populations that were not relevant to this study. In brief, limitations of this technique are

- Limited accessibility of very thick or highly pigmented organs.
- Potential phototoxicity affecting cell populations that were not analyzed in this study.

**Table 2:** Troubleshooting guide.

Step	Problem	Possible reason	Solution
Anesthesia	Mice react to footpad pinching	Anesthesia underdosed	Slowly add small doses of anesthetic intravenously until desired anesthetic depth is achieved
	
	Mice take longer than 1 h to recover from anesthesia	Anesthesia overdosed	Do not use more anesthesia than needed to reach desired anesthetic depth
		
		Low body temperature	Monitor the body temperature with a rectal probe. Place the animal on a heating pad during anesthesia recovery

Lethality up to 24 h after operation despite recovery from anesthesia	Postoperative ileus (a postoperative ileus can be detected by widened bowel loops becoming apparent through the ventral skin of the mouse)	Rough handling of the bowel	Handle bowels gently and with care. Use pre-wetted cotton swabs to handle the bowels. Refrain from pinching the bowel with forceps. Do not apply pressure or tension to the bowel. Keep bowels lubricated at all times
		
		Bowels were not placed back into the peritoneal cavity in the right organization	Follow Figure 1D for placing back the intestinal loops. The intestinal loop organization may vary to some extend individually
	
	Peritonitis	Too intensive light exposure leading to necrosis which in turn leads to intestinal perforation	Reduce UV illumination intensity or exposure time to UV illumination. Carefully lubricate the tissue to avoid heat development
		
		Introduction of infectious agents during the operation procedure	Work aseptically according to (37)
		
		Introduction of infectious agents after wound closure	Suture both the peritoneum and the skin sufficiently

Cell detection	Only dead cells are detected in flow cytometry	Contaminating digestive enzymes or intestinal contents in the cell suspension after isolation	Wash the pellets of isolated cells with PBS one or two times after obtaining single-cell suspension
		
		Too intensive light exposure leading to apoptosis and necrosis	Reduce UV illumination intensity or exposure time of UV illumination. Carefully lubricate the tissue to avoid heat development
	
	No converted protein signal can be detected	Insufficient UV light exposure for photoconversion	Increase duration and/or intensity of UV illumination
		
		Incorrect wavelength of converting light	The Dendra2 protein converts optimally with 405 nm wavelength illumination
		
		Insufficient excitation of red protein form	Use a flow cytometer equipped with a 561 nm laser
			
			Increase the sensitivity of the PMT detecting the red fluorescent signal
		
		Photoconverted protein diluted out by proliferation	Measure at earlier time points after photoconversion
	
	Converted cells are detected in the intestine 0 h after photoconversion	Photoconversion is not confined to the Peyer’s patches	Reduce the size of the tin foil stencil
		
		Peyer’s patch tissue is included in small intestinal sample	Note down the position of converted patches during the conversion procedure. Carefully remove all Peyer’s patches from the intestine before tissue digestion
			
			Discard the tissue closely surrounding the Peyer’s patches

Notably, we found that even immediately after photoconversion, single converted cells can be detected in the mesenteric lymph nodes. This was surprising given that the two organs are far apart. We postulate that these cells may have resided in the efferent lymphatics of one of the first converted Peyer’s patches, and had traveled to the mesenteric lymph nodes within the 30–40 min of photoconversion and blood collection. Such rapid migration is entirely realistic assuming a lymph flow rate of 100 µm/s (38).

Our photoconversion protocol generated a high percentage of converted cells (90%). This is considerably higher than when the intestinal lymphocytes were converted endoscopically (30%) or where cells in cervical lymph nodes were converted through the skin (46%) (24). The numbers are comparable to another study converting Dendra2 in the Peyer’s patches (99%). In contrast to our study, their protocol included much shorter but more intense ultraviolet light exposure (27).

We conclude that photoconversion of highly proliferative alloreactive T cells in the Peyer’s patches as presented in this protocol is a suitable and effective tool for studying physiological trafficking of T cells during inflammatory disease conditions such as acute GvHD. This protocol can be directly applied to the study of T cells under physiological steady state settings, in cancer models or under infectious conditions, to investigate cell migration. With some adjustments, it can also be applied to other cell types or to other animal models.

## Ethics Statement

This study was carried out in accordance with the recommendations and regulations of the Regierung von Unterfranken. The protocol was approved under the protocol number 55.2 2532-2-48.

## Author Contributions

KJ designed, planned, and carried out the experiments, analyzed and interpreted the data, and wrote the manuscript. ZM analyzed and interpreted the cell migration tracks. LS, JH, and ST helped in the photoconversion and cell isolation. D-DL, HS, MR, and MQ helped in cell isolation. KH helped with the establishment of ultraviolet illumination and gave feedback on the project. AB designed the study, interpreted the data, and revised the manuscript. All authors read and discussed the manuscript.

## Conflict of Interest Statement

The authors declare that the research was conducted in the absence of any commercial or financial relationships that could be construed as a potential conflict of interest.

## Supplementary Material

Figure S1 Scheme displaying the collection of blood from the vena cava. After ventral opening of the mouse, the intestines were laid to the right and the vena cava was exposed. A 30 G syringe containing 200 µl of lysis buffer was inserted at the junction of the left renal vein with the inferior vena cava and blood is slowly collected from the vessel.

Figure S2 Comparison of donor T cell yield from EDTA wash fraction and subsequent enzymatic digestion of the tissue. **(A)** Representative flow cytometry plots showing the percentage of donor cells within live single lymphocyte population. All donor cells in the intestine are washed out by the extensive washing steps (left), and no T cells are found after digesting the remaining tissue (right). **(B)** Quantification of donor T cells in the two fractions.

Figure S3 Exemplary flow cytometry gating scheme to identify the percentage of photoconverted cells among live donor lymphocytes. From left to right: lymphocytes are gated by size and granularity, live cells are gated negative for dead stain, and single cells were selected by plotting height and area of the forward scatter. Donor cells were identified by being positive for the green and/or the red fluorescent Dendra2 protein, and percentages of cells with red Dendra2 fluorescence were quantified within this population.
