# Supplementary figures and images for: Photoconversion of Alloreactive T Cells in Murine Peyer’s Patches During Acute Graft-Versus-Host Disease: Tracking the Homing Route of Highly Proliferative Cells In Vivo
Source: Front Immunol. 2018 Jun 27;9:1468. doi: 10.3389/fimmu.2018.01468 (PMC6036264; doi:10.3389/fimmu.2018.01468)

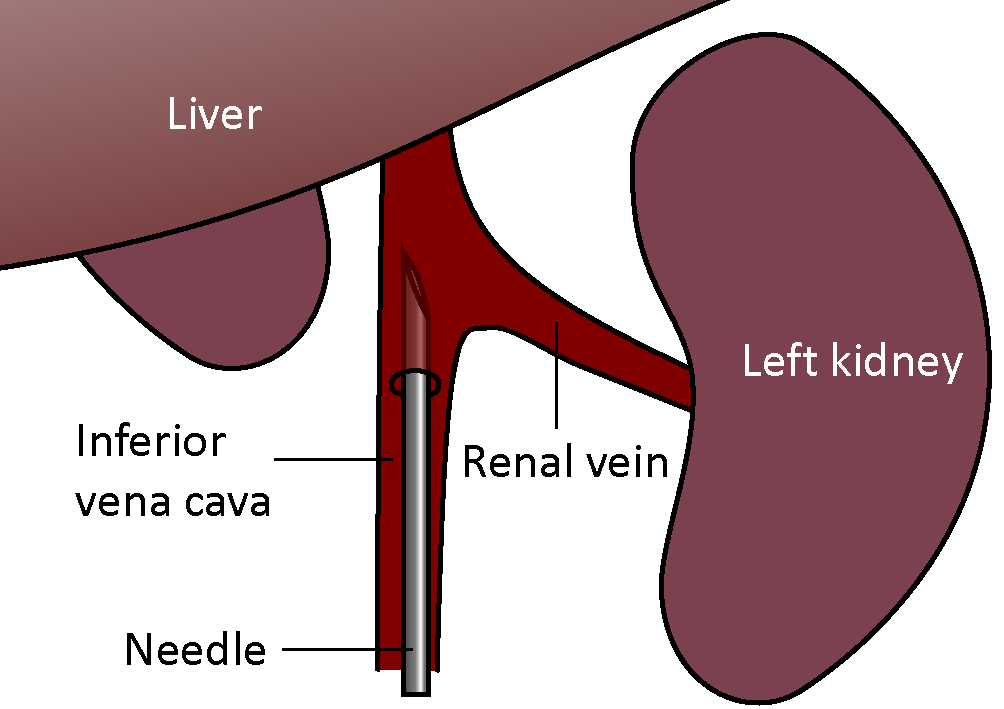

Supplement: Figure S1 — Scheme displaying the collection of blood from the vena cava. After ventral opening of the mouse, the intestines were laid to the right and the vena cava was exposed. A 30 G syringe containing 200 µl of lysis buffer was inserted at the junction of the left renal vein with the inferior vena cava and blood is slowly collected from the vessel. [file image_1.tif]

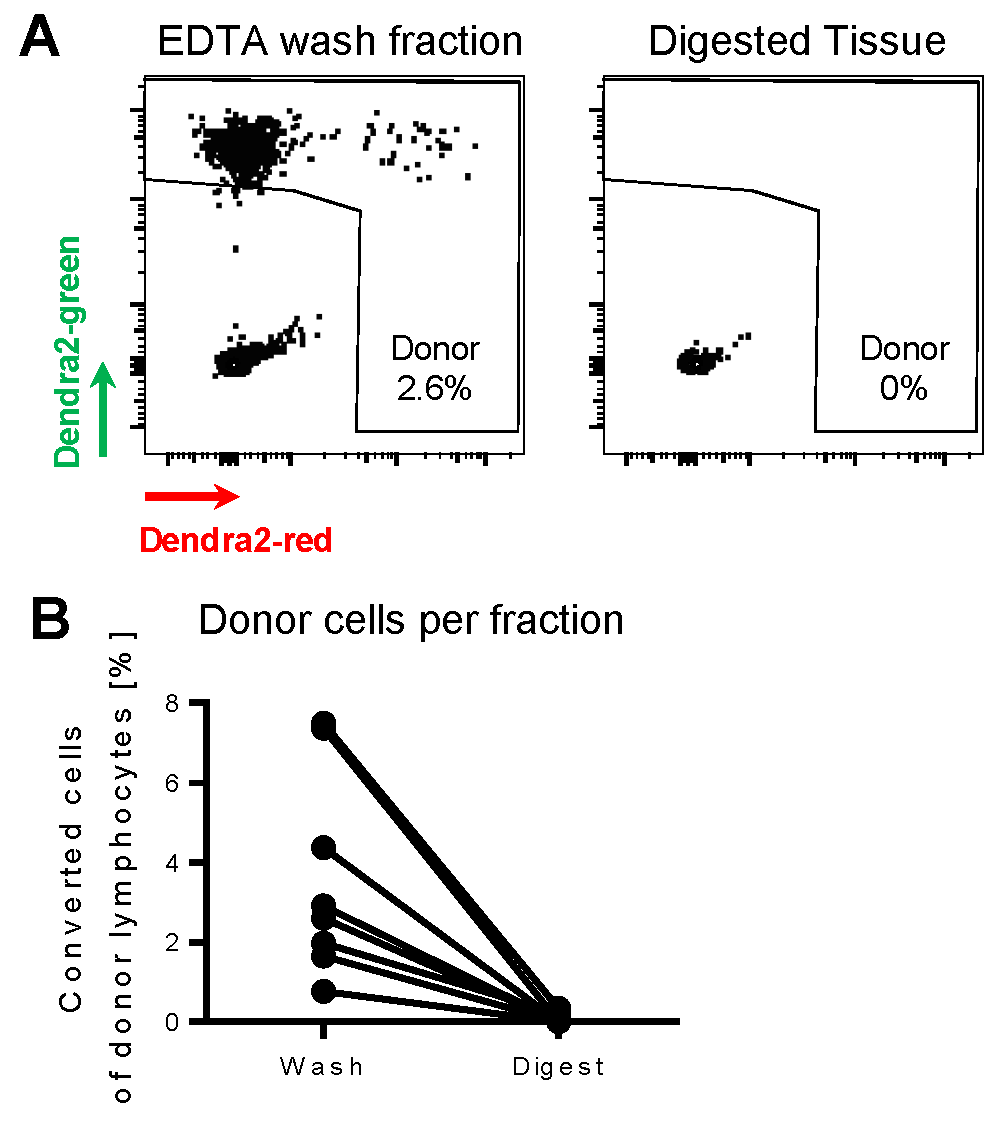

Supplement: Figure S2 — Comparison of donor T cell yield from EDTA wash fraction and subsequent enzymatic digestion of the tissue. (A) Representative flow cytometry plots showing the percentage of donor cells within live single lymphocyte population. All donor cells in the intestine are washed out by the extensive washing steps (left), and no T cells are found after digesting the remaining tissue (right). (B) Quantification of donor T cells in the two fractions. [file image_2.tif]

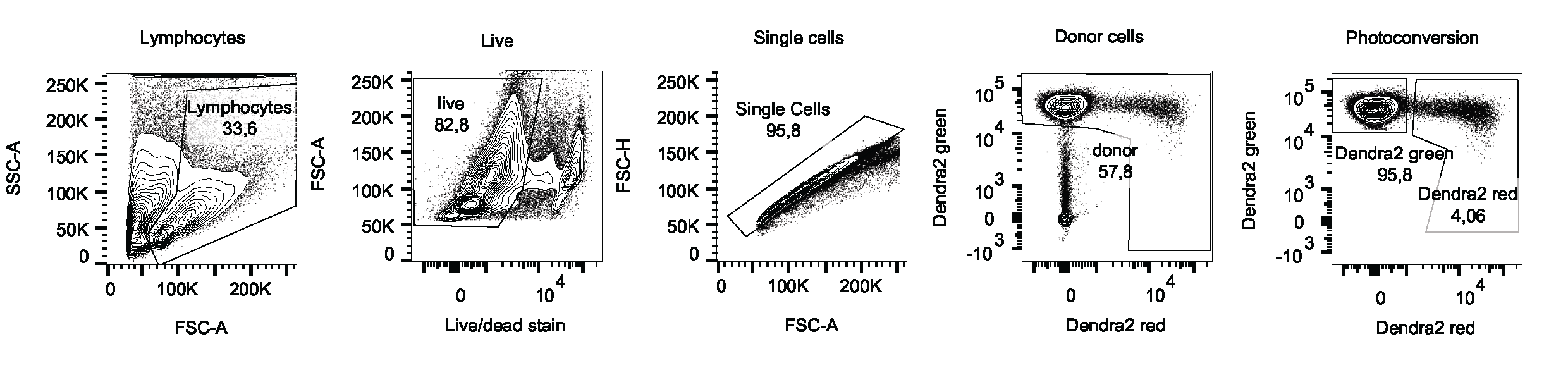

Supplement: Figure S3 — Exemplary flow cytometry gating scheme to identify the percentage of photoconverted cells among live donor lymphocytes. From left to right: lymphocytes are gated by size and granularity, live cells are gated negative for dead stain, and single cells were selected by plotting height and area of the forward scatter. Donor cells were identified by being positive for the green and/or the red fluorescent Dendra2 protein, and percentages of cells with red Dendra2 fluorescence were quantified within this population. [file image_3.tif]
