# Supplementary material for: Photoconversion of Alloreactive T Cells in Murine Peyer’s Patches During Acute Graft-Versus-Host Disease: Tracking the Homing Route of Highly Proliferative Cells In Vivo
Source: Front Immunol. 2018 Jun 27;9:1468. doi: 10.3389/fimmu.2018.01468 (PMC6036264; doi:10.3389/fimmu.2018.01468)
Supplement: Supplementary file 4 [file table_1.docx]

**Supplementary Table 1.** Representative compensation matrix for viability dye and Dendra2 protein.

|  | into Pacific blue | into FITC | into PE |
| --- | --- | --- | --- |
| from Pacific blue | - | 0,10% | 0,20% |
| from FITC | 0% | - | 46,50% |
| from PE | 0% | 0%-20% * | - |
